# Helicopter emergency medical services (HEMS) response to out-of-hospital cardiac arrest

**DOI:** 10.1186/1757-7241-21-1

**Published:** 2013-01-07

**Authors:** Richard M Lyon, Magnus J Nelson

**Affiliations:** 1 Emergency Medicine & Pre-hospital Care, Kent, Surrey, Sussex Air Ambulance, Redhill Aerodrome, Redhill, Surrey RH1 5YP, England; 2 Emergency Medicine, Royal Sussex Hospital, Brighton, England

**Keywords:** Cardiac arrest, Resuscitation, Emergency medical services, Helicopter emergency medical services

## Abstract

**Background:**

Out-of-hospital cardiac arrest (OHCA) is a common medical emergency with significant mortality and significant neurological morbidity. Helicopter emergency medical services (HEMS) may be tasked to OHCA. We sought to assess the impact of tasking a HEMS service to OHCA and characterise the nature of these calls.

**Method:**

Retrospective case review of all HEMS calls to Surrey and Sussex Air Ambulance, United Kingdom, over a 1-year period (1/9/2010-1/9/2011). All missions to cases of suspected OHCA, of presumed medical origin, were reviewed systematically.

**Results:**

HEMS was activated 89 times to suspected OHCA. This represented 11% of the total HEMS missions. In 23 cases HEMS was stood-down en-route and in 2 cases the patient had not suffered an OHCA on arrival of HEMS. 25 patients achieved return-of-spontaneous circulation (ROSC), 13 (52%) prior to HEMS arrival. The HEMS team were never first on-scene. The median time from first collapse to HEMS arrival was 31 minutes (IQR 22–40). The median time from HEMS activation to arrival on scene was 17 minutes (IQR 11.5-21). 19 patients underwent pre-hospital anaesthesia, 5 patients had electrical or chemical cardioversion and 19 patients had therapeutic hypothermia initiated by HEMS. Only 1 post-OHCA patient was transported to hospital by air. The survival to discharge rate was 6.3%.

**Conclusion:**

OHCA represents a significant proportion of HEMS call outs. HEMS most commonly attend post-ROSC OHCA patients and interventions, including pre-hospital anaesthesia and therapeutic hypothermia should be targeted to this phase. HEMS are rarely first on-scene and should only be tasked as a first response to OHCA in remote locations. HEMS may be most appropriately utilised in OHCA by only attending the scene if a patient achieves ROSC.

## Background

Out-of-hospital cardiac arrest (OHCA) is a common cause of mortality and serious neurological morbidity [1]. Efforts to reduce mortality from OHCA focus on the ‘chain-of-survival’. Rapid clinical intervention is required if the OHCA patient is to achieve return-of-spontaneous circulation.

Survival from OHCA is largely dependent on pre-hospital events. Survival with good neurological outcome is influenced by good quality cardio-pulmonary resuscitation (CPR) [2] and early defibrillation. Both of these interventions are provided by all ground-based ambulance units in the United Kingdom. Transport with on-going CPR rarely has a positive outcome in OHCA [3]. Advanced interventions, such as intubation, cannulation and sedation/anaesthesia, have not yet been shown to influence outcome from OHCA [4]. The need for delivering advanced critical care early to the OHCA patient is much debated.

In several countries, helicopter emergency medical services (HEMS) are tasked to attend OHCA incidents. HEMS can provide a rapid response to the scene, particularly in rural or difficult to access locations. HEMS often carry a pre-hospital doctor as part of the team who is frequently capable of administering pre-hospital anaesthesia, advanced airway interventions, inotropic support and initiating therapeutic hypothermia. Advanced post-ROSC care can be initiated pre-hospital by HEMS and the patient transported rapidly, possibly for early percutaneous coronary intervention, to hospital, particularly in cases of ST-elevation myocardial infarction [5]. The impact of HEMS responding to OHCA has yet to be fully evaluated.

Surrey-Sussex HEMS provides advanced pre-hospital care to the southeast of England. Two helicopters, located at two separate bases provide pre-hospital medical and trauma care to three counties (population approximately 2.5 million) and undertakes approximately 600 missions per year. Approximately 60% of missions are for trauma and 40% for acute medical illness. The incidence of OHCA is approximately 40 per 100,000 annually. The HEMS team consists of a pilot, a pre-hospital doctor, from an anaesthesia or emergency medicine background, and a critical care paramedic. Cold intravenous saline is carried in a bespoke cool box to initiate therapeutic hypothermia in all patients who achieve return of spontaneous circulation, in whom there is no contraindication to cooling. The HEMS team can reach any point in the counties within 20 minutes of dispatch. HEMS can currently be activated to all cases of OHCA, although were death is suspected (rigor mortis, post-mortem staining, injury incompatible with life) the dispatcher may wait for further information from a land ambulance crew prior to activating HEMS. We sought to assess the impact of tasking a HEMS service to OHCA and characterise the nature of these calls.

## Methods

A retrospective case review of all missions conducted by Surrey-Sussex HEMS over a one-year period (1/9/2010-1/9/2011) was conducted. All missions were reviewed and those with medical OHCA as the initial dispatch were selected. Inclusion criteria were cases were HEMS attended an OHCA of non-traumatic aetiology. Exclusion criteria were HEMS stand-downs and case where the patient had not suffered a cardiac arrest or the OHCA was felt to be traumatic in origin.

HEMS is activated by a designated HEMS paramedic in the ambulance dispatch centre. The HEMS paramedic was the ability to screen all calls in real time and also listen to select calls if needed. Specific dispatch criteria exist for tasking HEMS to trauma incidents but tasking to medical incidents is at the discretion of the HEMS paramedic.

The ambulance dispatch log, HEMS patient record sheet and patient vital signs summary were all reviewed. Utstein data was reviewed for each patient and collated on a Microsoft Excel (Microsoft Inc) database. Descriptive statistics were used to describe the study cohort with median and interquartile range used for continuous variables. The study was registered as a service evaluation and formal ethical approval waived. Patients were followed up to hospital discharge.

## Results

### Cases

During the one-year research period, Surrey-Sussex HEMS attended 636 missions. Of these, 89 (14%) were to suspected OHCA. In 23 (26%) cases HEMS was stood down en-route to scene by the land ambulance crew. In these cases the patient was either not in cardiac arrest on arrival of the ambulance or pronounced dead by the land ambulance crew. In two cases (2%) the HEMS crew attended a scene and found a patient who had not suffered a cardiac arrest.

Overall, 64 OHCA HEMS missions were included. The patient was male in 49 (77%) cases. Patient age ranged from 3 months to 87 years (mean= 54 years). There were 3 cases of paediatric (<16 years) OHCA.

The location of OHCA was a private residence in 26 (41%) cases, public area in 34 (53%) cases and not documented in 4 (6%) cases. 39 (61%) cases were witnessed and 25 (39%) cases unwitnessed. Bystander CPR was performed in 45 (70%) cases, not performed in 11 (17%) cases and not documented in 9 (14%) cases.

The initial cardiac rhythm was ventricular fibrillation in 36 (56%) cases, pulseless electrical activity in 14 (22%) cases and asystole in 14 (22%) cases.

### Response

The average response time to suspected OHCA calls by land ambulance in Surrey and Sussex is less than 8 minutes with the first ambulance dispatched within 2 minutes of the initial emergency call in the majority of cases. The median time from initial emergency call to HEMS activation was 11 minutes (IQR 6–22 mins). The median response time from HEMS activation to HEMS arriving with the patient was 17 minutes (IQR 11–21 mins). The median time from initial emergency call to HEMS arriving with the patient was 31 minutes (IQR 22–40 mins). The median time from emergency call to arrival at hospital was 90 minutes (IQR 79–101 mins).

### Resuscitation

In total, 25 (39%) patients achieved ROSC at-scene, 13 (20%) achieved ROSC prior to HEMS arrival and 12 (19%) post-HEMS arrival. Of the paediatric cases, 2 patients achieved ROSC, 1 did not and all were transported to hospital.

All patients who achieved ROSC were sedated, paralysed and ventilated following HEMS arrival. 4 patients who achieved ROSC required drug-assisted rapid sequence induction (RSI).

**Figure 1:**
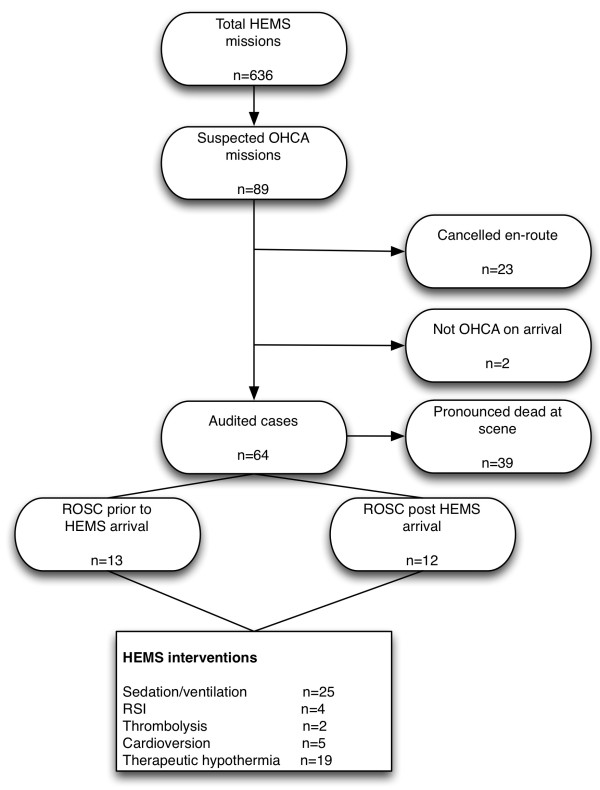
**Summary of helicopter emergency medical services missions to out-of-hospital cardiac arrests and clinical interventions performed. **HEMS: helicopter emergency medical services, OHCA: out-of-hospital cardiac arrest, ROSC: return of spontaneous circulation, RSI: rapid sequence induction.

HEMS thrombolysed two patients pre-hospital, both for suspected massive pulmonary embolism. HEMS performed chemical/electrical cardioversion in 5 patients for unstable broad complex tachycardias. Therapeutic hypothermia was initiated in 19 post-ROSC patients using cold intravenous saline and target temperature (<34**°**C) was achieved prior to hospital arrival in 15 patients. A summary of the OHCA call and interventions performed by HEMS is shown in Figure 1.

All patients who achieved ROSC (n=25) were transported to hospital, 24 by land ambulance and 1 by air ambulance. Only 2 (3%) patients re-arrested during transport to hospital and both were being transported by land. Of the post-ROSC patients transported, 9 were taken directly for percutaneous coronary intervention following a post-ROSC 12-lead ECG suggestive of a primary coronary occlusive origin. Of the patients seen by HEMS, 4 (6.3%) survived to discharge from hospital.

## Discussion

We found the HEMS response to OHCA to be predominantly for post-ROSC patients. In our setting, the interventions (CPR and defibrillation) were delivered by ambulance ground units in all cases of OHCA prior to HEMS arrival. In the majority of cases in our region, OHCA are attended by paramedics capable of performing Advanced Life Support, including intubation. The paramedics are not currently permitted to administer anaesthetic agents, muscle paralysis or initiate cooling. In geographical settings such as south east England, any delay in activating HEMS will make ground units more likely to be first on-scene, as shown in this case series. Except where geographical location may present a challenge or delay emergency medical response, HEMS activation to OHCA should be to deliver advanced post-ROSC care. Provided there is early, competent land-based emergency medical intervention, activation or landing at the scene of an OHCA, with the associated risks to medical crew, public and aircraft, may be best reserved for OHCA patients who have achieved ROSC. In our study HEMS were never the first emergency medical response to reach an OHCA patient. There are theoretical benefits of HEMS providing early critical care intervention, such as pre-hospital anaesthesia and initiating prehospital therapeutic hypothermia but further research is warranted to evaluate the impact of these interventions on outcome.

Landing a helicopter at the scene of OHCA is not without risk. In systems were ground and air-based emergency medical services operate together, it may be appropriate for direct communication to occur between the ground and air teams, with HEMS only attending the scene in cases of ROSC, unless a specific HEMS intervention is required.

Previous studies examining the role of HEMS in OHCA have come to similar conclusions. Skogvoll et al. suggested HEMS may contribute to improved survival through enhanced post-ROSC care [6]. Hospital studies [7] have demonstrated the benefit of a standardised post-ROSC treatment protocol, which could be adopted by HEMS and commenced earlier in the patient’s care pathway. Where the transport distance to hospital is long, HEMS can provide advanced critical care, including therapeutic hypothermia, in a timely fashion [8].

There is little evidence to support transporting OHCA patients by air who remain in cardiac arrest, except in special circumstances, for example hypothermic patients. Quality of cardiopulmonary resuscitation has been shown to be poor during aeromedical transport [9], but the use of mechanical CPR devices may be considered and warrants further research.

Post-ROSC, HEMS can facilitate rapid transport for PCI, particularly if the ground transport time is long. HEMS transport of post-ROSC OHCA patients has previously been suggested to improve outcome [10] but this is likely to be dependent on the aetiology of cardiac arrest, geography and transport times involved. In the paper published by Werman et al., a significant number of patients had non-cardiac cause of OHCA compared to our study. Whether early, advanced medical intervention is beneficial or whether the rapid transport is more beneficial warrants further research. Rapid transport by air for coronary intervention is warranted to meet optimum angiography time windows for patients with acute coronary syndromes requiring immediate coronary intervention [5,11]. As the incidence of re-arrest is low, helicopter transport should be considered in cases of remote geography. In our region, the fastest means if transporting the patient to hospital is often via land ambulance.

This study has several limitations, namely the retrospective nature of the case review and the relatively small number of patients included. It was not possible to quantify the impact of specific HEMS interventions on patient outcome. However, the results suggest a clear outcome that HEMS is likely to only be beneficial to post-ROSC patients, except in a few select cases. In the absence of any formal OHCA dispatch criteria, HEMS are also likely to be dispatched to a select subgroup of OHCA. These are likely to include witnessed, young age, high prevalence of VF as the presenting rhythm or OHCA occurring in remote locations.

## Conclusion

OHCA represents a significant proportion of HEMS call outs. HEMS most commonly attend post-ROSC OHCA patients and interventions, including pre-hospital anaesthesia and therapeutic hypothermia should be targeted to this phase. HEMS are rarely first on-scene and should only be tasked as a first response to OHCA in remote locations. OHCA patients in this study rarely re-arrested and transport to hospital by air should not be precluded by fear of re-arrest. HEMS may be most appropriately utilised in OHCA by only attending the scene if a patient achieves ROSC.

## Abbreviations

HEMS: Helicopter emergency medical services; IQR: Inter-quartile range; OHCA: Out-of-hospital cardiac arrest; PCI: Percutaneous coronary intervention; ROSC: Return-of-spontaneous circulation; RSI: Rapid sequence induction.

## Competing interests

The authors declare that they have no competing interests.

## Authors’ contributions

Both authors contributed to data collection, analysis and manuscript preparation. Both authors read and approved the final manuscript.
